# A Rare Case Report of Fatal Fulminant Hepatic Failure in a Child due to Mixed *vivax* and *falciparum* Infection

**DOI:** 10.1155/2011/614054

**Published:** 2011-10-19

**Authors:** Neha Thakur, Ravitanaya Sodani, J. Chandra, Deonath Mahto

**Affiliations:** Department of Pediatrics, Kalawati Saran Children Hospital, New Delhi 110001, India

## Abstract

Malaria remains an overwhelming problem in the tropical developing countries, with 300 to 500 million new cases and about a million deaths per year (Mishra et al., 2003). Malaria is a potentially life-threatening disease in the tropics. Jaundice is one of the severe manifestations of *falciparum* malaria. Its incidence (Mishra et al., 2003). varies between 10 and 45% in different reports and is seen more in adults than in children. Jaundice may vary from mild to very severe. However, clinical signs of hepatic encephalopathy (such as liver flaps) are never seen unless there is presence of concomitant viral hepatitis (WHO, 2000). Our case is a 6-year-old female child presented with fever, jaundice, and anasarca. Peripheral smear showed trophozoites and schizonts of *Plasmodium* (*P.*) *vivax* and trophozoites and gametocytes of *P. falciparum.* Viral markers for hepatitis were negative. She developed fulminant hepatic failure and expired after 26 hours of admission.

## 1. Background

Malaria remains a major problem in many parts of the world. Approximately 500 million people are affected annually, and approximately 3 million, mostly children, die of *falciparum* malaria each year [1, 2]. According to the World Health Organization's criteria, the recognition of one or more of the following clinical features should raise the suspicion of severe malaria: cerebral malaria (coma), severe anemia (haemoglobin <5 g/dL), renal failure (serum creatinine >3 mg/dL), pulmonary oedema, hypoglycaemia (glucose <40 mg/dL), shock, disseminated intravascular coagulation, repeated generalized convulsions, acidosis (pH <7.25), macroscopic haemoglobinuria, hyperparasitaemia (>5 percent of the erythrocytes infested by parasites), or jaundice (bilirubin >3 mg/dL). A number of patients with malaria develop severe manifestations, and these patients require urgent and intensive care. In patients with severe malarial infestation, the incidence of jaundice is reported to be 2.58% only [3]. Jaundice in severe *P. falciparum* malaria is multifactorial (intravascular haemolysis of infested RBCs/normal RBCs, possibly micro-angiopathic haemolysis associated with DIC, hepatic dysfunction, associated haemoglobinopathies (not uncommon in malaria-prone areas), drug-induced haemolysis, and G6PD deficiency). Hepatic dysfunction in malaria has been known for many years, but hepatic encephalopathy is unusual. According to the World Health Organization (WHO), apart from jaundice, signs of hepatic dysfunction are unusual. Jaundice has been found to be more common in *falciparum* as compared to *vivax* malaria. Hazra et al. found an association of jaundice in 40% and 9.09% of cases with *P. falciparum *and *P. vivax*, respectively, from Calcutta [4]. Echeverri et al. in a study of *vivax* malaria from Colombia have reported an incidence of jaundice in 15% [5]. Seth et al. found jaundice in 7.7% of cases of *falciparum* malaria while they did not find jaundice in *vivax* malaria [6]. There has not been a single case report of fulminant hepatic failure due to mixed malarial infection in children. Jaundice in children infected with malarial parasite is reported but its incidence is less compared to adults. According to the WHO, clinical signs of liver failure such as asterixis are almost never seen unless there is concomitant viral hepatitis. Fulminant hepatic failure (FHF), defined as the onset of encephalopathy within 8 weeks of the first symptom of hepatic injury, is commonly due to viral hepatitis or drug use. Infectious diseases such as sepsis or typhoid fever may present rarely with FHF. Severe malaria, defined as jaundice or renal failure with or without altered consciousness, is more common in adults than children and clinically may resemble acute liver failure. Our case presented with fulminant hepatic failure due to mixed malarial infection, and concomitant viral hepatitis was ruled out.

## 2. Case Presentation

Six-year-old female child, first product of nonconsanguineous marriage resident of New Delhi, presented with fever for four days, yellowish discoloration of eyes, and urine for two days, progressive swelling of whole body starting from feet for one day. Fever was moderate to high grade, intermittent in type associated with chills and rigor subsided with medication and profuse sweating. She was having high coloured urine for two days. Progressive swelling of whole body for last one day starting from feet then involved the whole body. Stools were normal in colour. There was no history of black coloured stools, vomiting, bleeding from any site, and any change in conscious level, jaundice in the past, any history of blood transfusion, and intravenous medication in the past or any history of drug intake. There was no history suggestive of respiratory tract infections, skin infection, decreased micturition, and reddish coloured urine. There was no significant past, antenatal, perinatal, or family history. The child was fully immunized and was receiving adequate calories and proteins for her age.

On examination, the child was conscious, cooperative, and orientated. At admission her pulse was 78 per minute, respiratory rate 26 per minute, and blood pressure 98/68 mm of mercury. There were severe pallor, and icterus. Lymph nodes were not palpable. On abdominal examination liver was palpable 4 cms below costal margin with span of 10 cm the spleen was palpable 1 cms below costal margin. Her respiratory, cardiovascular (CVS), and central nervous system (CNS) examination was unremarkable.

On investigation haemoglobin was 4.9 g/dL, total leucocyte count 10.6 × 10^3^ per *μ*L with polymorphs 54%, lymphocytes 42%, monocyte 4%, platelet count 11×10^3^ per *μ*L and erythrocyte sedimentation rate 8 mm/1st hour by Westergren method. Blood sugar was 84 mg/dL. Total serum bilirubin was 27.5 mg/dL, indirect fraction of 18 mg/dL. SGOT was 495 and SGPT 210. Serum total protein was 2.8 gram (gm) and albumin 1 gm per 100 mL of blood. Blood urea nitrogen was 189 mg/dL, creatinine 0.6 mg/dL, sodium 136 meq/L, potassium 4.1 meq/L. Prothrombin time was 17.3/12 with INR of 1.44. Viral markers (HBsAg, IgM hepatitis C, A and E) were negative. Chest X-ray was normal and ultrasonography abdomen showed hepatosplenomegaly. On peripheral smear trophozoites and schizonts of *P. vivax* and trophozoites and gametocytes of *P. falciparum* were seen. Rapid malarial antigen test was positive. Arterial blood gas analysis showed pH 7.45, partial pressure (PP) of oxygen 104, PP of carbon dioxide 45, and standard bicarbonate 28 meq/L. Urine routine and microscopy were normal. She was given injection of Artesunate 2.4 gm per kilogram body weight, along with cefotaxime and vitamin K. She received fresh frozen plasma, packed red cell, platelets along with inotropes. Within five hours of admission she developed profuse upper gastrointestinal bleeding, along with deterioration of consciousness. She went into shock, then cardiorespiratory arrest from which she could not be revived.

## 3. Discussion

Malaria is a major health problem in many of the developing and tropical countries. Although rare, severe malaria must be distinguished from viral FHF. This can have profound implications for the line of treatment and ultimate patient outcome. The liver is the first organ to be affected in a case of *P. falciparum* malaria. After the initial stage (preerythrocyte schizogony), merozoites are released into the blood stream. They do not have exoerythrocytic schizogony. But in the patients of severe malaria, the liver may be involved to different extents. In patients with severe malarial infection, the incidence of jaundice is reported to be 2.58% only [3]. Presence of jaundice in *falciparum* malaria indicates a more severe illness with higher incidence of complications.

**Causes of Jaundice in Malaria**

Direct causes: malarial hepatitis, intravascular hemolysis of parasitized RBC, and septicemic hepatitis.
Indirect causes: microangiopathic hemolysis associated with DIC, G6PD-related hemolysis, and anti-malarial-drug induction.
Unrelated causes: coexisting acute viral hepatitis.

**Underlying Chronic Hepatitis**

Hyperbilirubinaemia is mostly of unconjugated type. Often jaundice is mild, and total serum bilirubin is below 5 mg/dL, but at times bilirubin rises beyond 50 mg/dL. Enzymes are usually raised (within 3 to 8 times); alanine transaminase (ALT) never reaches to the level of viral hepatitis. 5′ nucleotidases and GGT concentrations may be moderately elevated. Hypoalbuminaemia is also seen in these patients. Prothrombin time may be moderately prolonged. The literature on the hepatic involvement in malaria has largely shown severe infection with *P. falciparum* infection. There have been occasional reports of mixed infection with P. *vivax* [7] and hepatitis E [8] along with *P. falciparum*, resulting in malarial hepatitis. The management of malarial hepatitis is not different from the management of severe malarial infection. In endemic areas, it is important to have a high degree of suspicion of severe infection with malarial parasite presenting as acute febrile illness with hepatic dysfunction. The other conditions prevalent in tropical countries like acute viral infections leading to hepatitis, enteric fever and leptospirosis should be figured in the differential diagnosis. Since the clinical presentation may not differ much, laboratory help may be needed to establish the etiology of malarial hepatitis. Routine, single peripheral smear examination may not be sufficient, so repeated smears are required to pick up the diagnosis. Treatment of severe malaria is artesunate or quinine intravenously till the child becomes conscious thereafter given orally for a total of seven days.

##  Learning Points/Take-Home Message

- Jaundice is one of the common severe manifestations of *falciparum* malaria.
- Altered consciousness in malarial hepatitis, although uncommon, may make the distinction between malarial hepatitis and viral FHF difficult.
- Failure to recognize this uncommon presentation often leads to delayed diagnosis, resulting in poor outcome.
- Severe malaria due to *P. falciparum* may masquerade as FHF. Simple clinical and laboratory parameters can easily distinguish severe malaria from FHF.
- Early diagnosis of malarial FHF has profound implications because the cornerstone of therapy is quinine, whereas liver transplantation may be required for FHF in selected patients.
